# Audit of high dose antipsychotic prescribing in the havering community recovery team

**DOI:** 10.1192/bjo.2021.939

**Published:** 2021-06-18

**Authors:** Chinedu Umeh, Simona Ionita

**Affiliations:** North East London Foundation Trust

## Abstract

**Aims:**

The main aim of this audit was to determine the prevalence of HDAP in Havering Community Recovery Team (CRT). The secondary aim was to determine how well HDAP has been monitored and documented - specifically, whether discussions around the reasons for continuing and the risks and benefits have been discussed.

**Background:**

There is a focus to reduce high dose antipsychotic prescribing (HDAP) due to the lack of evidence that it is efficacious and that smaller doses have an equivalent effect and are better tolerated. Similarly, the consensus by the Royal College of Psychiatrists is that any prescribing of high dose antipsychotics should be an 'explicit, time-limited individual trial’ with a distinct treatment target. There should be a clear plan for regular clinical review including safety monitoring. The high-dose regimen should only be continued if the trial shows evidence of benefit that is not outweighed by tolerability or safety problems.' Following a CQC inspection in 2014 of NELFT which found that the trust was failing to comply with the relevant requirements of the Health and Social Care Act 2008 with regards to safe use of medicines, yearly audits of inpatient HDAP have been undertaken. Although improvements have been made in the inpatient setting, no such audits have been performed in the community setting and consequently there is no data in NELFT regarding community services compliance with the above regulations.

**Method:**

All 349 patients in Havering CRT clinical records were screen by either using RIO or GP letters from recent CPA reviews. A data collection and analysis tool was created using Microsoft Excel. Data collection and analysis was carried out by the project lead and checked by a fellow project member.

**Result:**

Of the 349 patients included for analysis:

- 16 (4.58%) of patients were prescribed a high-dose antipsychotic

Of the 16 prescribed high dose antipsychotics:

- 0 out of 16 had the high dose antipsychotic monitoring form available
- 12 (75%) had well documented evidence of review of HDAP.
- 4 (25%) had no documented evidence of review of HDAP.

**Conclusion:**

There is a small group of patients receiving high dose antipsychotic therapy for which better monitoring is needed. This should include education of staff regarding HDAP, better documentation in their care plans and working with pharmacy to make HDAP monitoring forms available widely in the community.

